# Comprehensive analysis of ubiquitin-proteasome system genes related to prognosis and immunosuppression in head and neck squamous cell carcinoma

**DOI:** 10.18632/aging.203411

**Published:** 2021-08-16

**Authors:** Juncheng Wang, Jianing Li, Luan Zhang, Yuexiang Qin, Fengyu Zhang, Rulong Hu, Huihong Chen, Yongquan Tian, Zhifeng Liu, Yuxi Tian, Xin Zhang

**Affiliations:** 1 Department of Otolaryngology Head and Neck Surgery, Xiangya Hospital, Central South University, Changsha 410008, Hunan, P.R. China; 2 Department of Clinical Research, Sun Yat-Sen University Cancer Center, State Key Laboratory of Oncology in South China, Collaborative Innovation Center for Cancer Medicine, Guangzhou 510060, Guangdong, P.R. China; 3 Department of Health Management, The Third Xiangya Hospital, Central South University, Changsha 410013, Hunan, P.R. China; 4 Department of Otorhinolaryngology, The First Affiliated Hospital of University of South China, Hengyang 421001, Hunan Province, P.R. China; 5 Otolaryngology Major Disease Research, Key Laboratory of Hunan Province, Changsha 410008, Hunan, P.R. China; 6 Department of Geriatrics, Respiratory Medicine, Xiangya Hospital, Central South University, Changsha 410008, Hunan, P.R. China

**Keywords:** head and neck squamous cell carcinoma (HNSCC), ubiquitin proteasome system (UPS), the cancer genome atlas (TCGA) database, gene expression omnibus (GEO) database, immunosuppression

## Abstract

The ubiquitin-proteasome system (UPS) with a capacity of degrading multiple intracellular proteins is an essential regulator in tumor immunosurveillance. Tumor cells that escape from recognition and destruction of immune system have been consistently characterized an important hallmark in the setting of tumor progression. Little know about the exact functions of UPS-related genes (UPSGs) and their relationships with antitumor immunity in head and neck squamous cell carcinoma (HNSCC) patients. In this study, for the first time, we comprehensively identified 114 differentially expressed UPSGs (DEUPSGs) and constructed a prognostic risk model based on the eight DEUPSGs (BRCA1, OSTM1, PCGF2, PSMD2, SOCS1, UCHL1, UHRF1, and USP54) in the TCGA-HNSCC database. This risk model was validated using multiple data sets (all *P* < 0.05). The high-risk score was found to be an independently prognostic factor in HNSCC patients and was significantly correlated with T cells suppression. Accordingly, our risk model can act as a prognostic signature and provide a novel concept for improving the precise immunotherapy for patients with HNSCC.

## INTRODUCTION

Head and neck squamous cell carcinoma (HNSCC) affects more than 800,000 people each year worldwide, leading patients with HNSCC about sixth in global incidence [1, 2]. HNSCC is a collection of malignancies with complex local autonomy, given that it originates from the squamous epithelium of the upper aerodigestive tract [3], attributing to this malignancy with high morbidity and mortality. Although advances in therapeutic strategies on local control have been improved, the 5-year overall survival (OS) rate remains 40%-50% [4, 5]. In this regard, there is a potential to identify novel biomarkers to broaden current treatment and enhance survival for patients with HNSCC.

The ubiquitin-proteasome system (UPS) is responsible for the degradation of more than 80% of intracellular proteins, such as short-lived and misfolded or non-essential proteins [6, 7]. UPS is involved in multiple biological functions, including gene transcription, translation and repair, cell cycle, cell proliferation, apoptosis, and antigen presentation [8, 9]. As UPS is involved in multitudinous critical functions, dysfunction of UPS may result in various diseases, such as nervous system diseases and cancers [10–14]. The UPS is a complex system encompassing a huge number of genes, including those encoding 10 ubiquitin (Ub)-activating enzymes (E1s), approximately 40 Ub-conjugating enzymes (E2s), over 600 validated Ub-protein ligases (E3s), approximately 100 deubiquitinating enzymes (DUBs), and nearly 50 proteasome subunits [15–18]. Accumulating studies have demonstrated that aberrant expression of UPS members altered proteolysis, facilitating the tumorigenesis and progression of HNSCC [19]. For instance, UBE2C (ubiquitin-conjugating enzyme E2 C), an E3s that overexpressed in various cancers and served as an inhibitor of p53 by facilitating its ubiquitination-mediated degradation. It is an agonist of ZEB1/2, ABCG2, and ERCC1 by promoting their transcript, which played an essential role in increasing cell proliferation and invasion, inducing epithelial-mesenchymal transitions (EMT), and chemotherapeutic resistance [20–23]. In addition, abnormal expression of PSMD2 (proteasome 26S subunit ubiquitin receptor, non-ATPase 2), a subunit of the 19S regulatory particle (RP) of the proteasome, has been reported in lung adenocarcinoma, breast cancer, and hepatocellular carcinoma [24–26]. The clinical significance of UPS genes has not been systematically investigated in patients with HNSCC. Therefore, the mechanism underlying the relationship between UPS-related genes (UPSGs) and the prognosis of HNSCC patients still needs to be further determined.

The Gene Expression Omnibus (GEO) and The Cancer Genome Atlas (TCGA) provide potent resources for directly obtaining gene expression profiles from patient tissues. Therefore, this study aims to provide a systematic investigation of the expression patterns via bioinformatics analysis and a prognostic risk model based on the UPSGs. We proposed that UPSGs are associated with the prognosis of patients with HNSCC. Importantly, our finding demonstrated that HNSCC patients with poor prognoses have high-risk scores that are associated with impaired T cell antitumor responses.

## RESULTS

### Identification of differentially expressed UPSGs in HNSCC tissues

**Figure 1:**
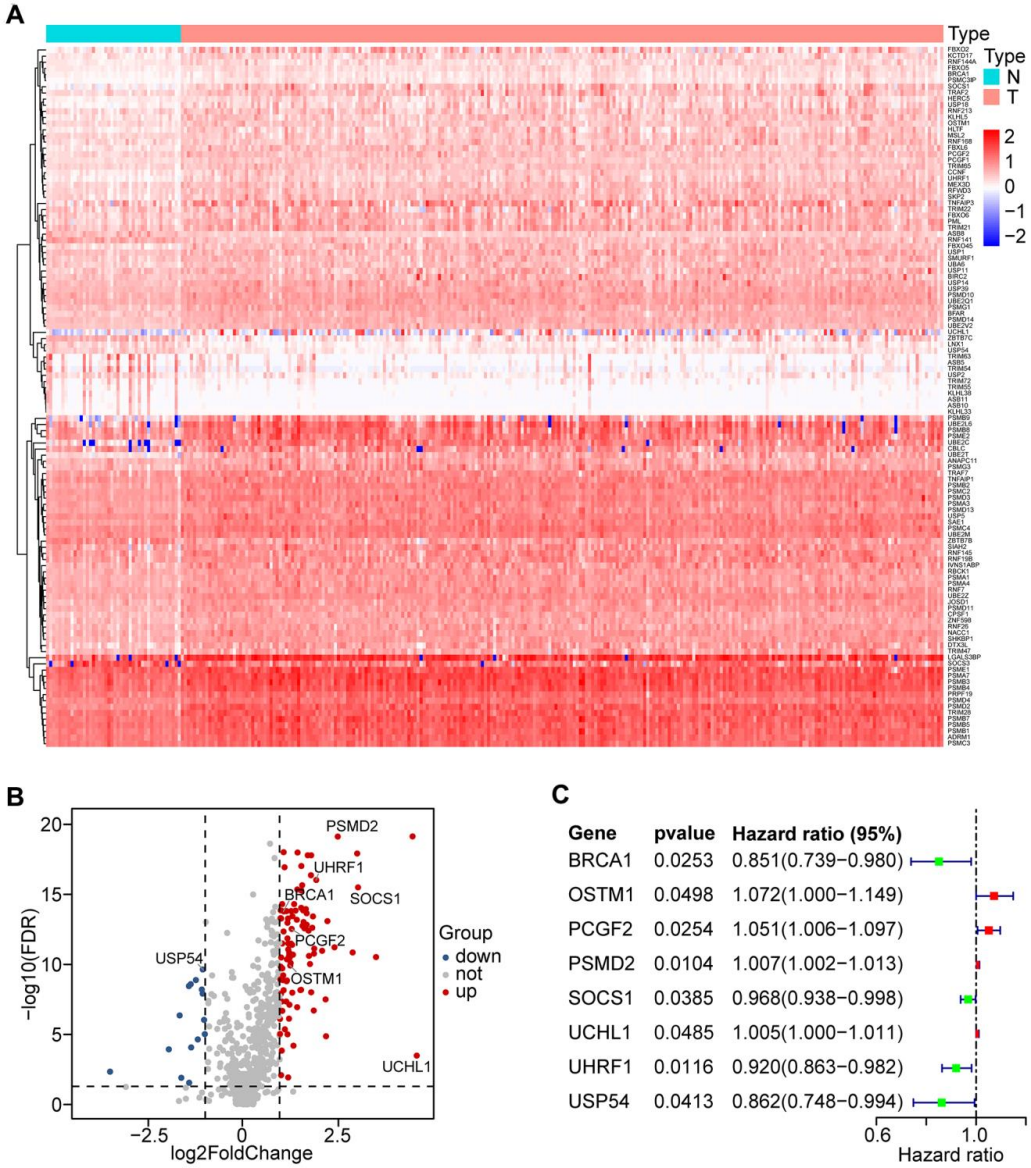
**Differential expression of UPS-related genes (UPSGs) and identification of 8 UPSGs with prognostic value in HNSCC samples.** (**A**) 114 differentially expressed UPSGs (DEUPSGs) are depicted as a heat map. (**B**) 97 upregulated and 17 downregulated DEUPSGs are shown as a volcano plot (FDR < 0.05 and |Fold change| > 1.5). (**C**) The eight risk DEUPSGs in the prognostic risk model are shown using a forest plot.

We analyzed the expression of 804 UPSGs (Supplementary Table 1) that were distributed through all chromosomes in 498 head and neck tumor tissues and 44 adjacent tissues. 114 differentially expressed UPSGs (DEUPSGs) were identified, as shown in Supplementary Figure 1, where 97 upregulated and 17 downregulated DEUPSGs were observed (FDR < 0.05 and |Fold change| > 1.5, Figure 1A, 1B). The genomic information of DEUPSGs was shown in Supplementary Table 2.

### Functional analysis of DEUPSGs in HNSCC

**Figure 2:**
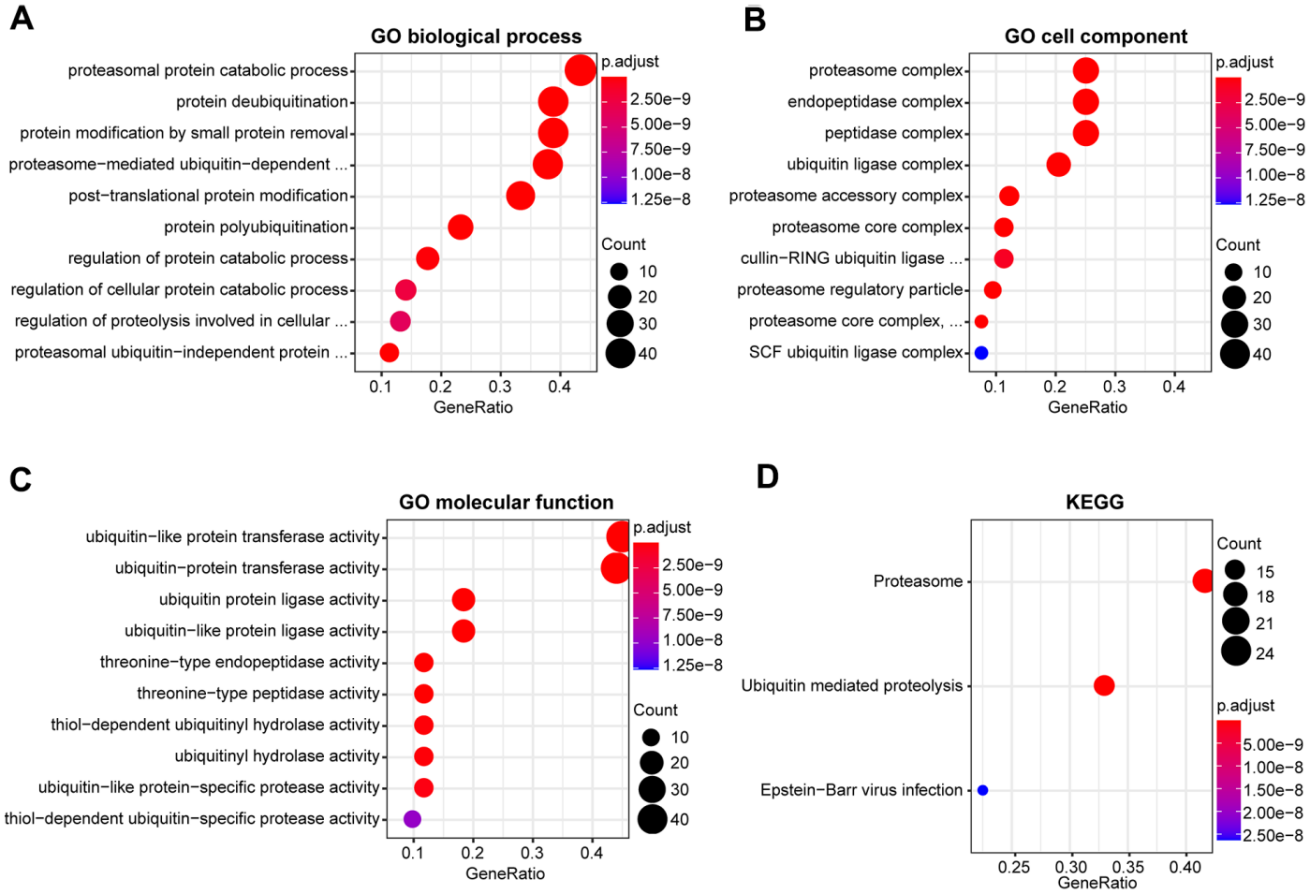
**Functional enrichment analysis of DEUPSGs in HNSCC.** (**A**–**C**) The top ten enriched terms in the GO analysis belonged to biological process (**A**), cell component (**B**), and molecular function (**C**) for DEUPSGs are demonstrated using an enriched scatter diagram. (**D**) The enriched pathways of the KEGG pathway analysis are showed using a scatter diagram. GO, gene ontology; KEGG, Kyoto Encyclopedia of Genes and Genomes.

To assess the potential functions of DEUPSGs, Gene Ontology (GO) and Kyoto Encyclopedia of Genes and Genomes (KEGG) pathway were analyzed using the TCGA database. The most significant GO terms were enriched for determining DEUPSGs functions, with respect to biological process (BP), cellular component (CC), and molecular function (MF), were shown in Figure 2A–2C. Enriched terms were significantly correlated with the proteasomal protein catabolic process, proteasome complex, and ubiquitin-like protein transferase activity. The KEGG pathway enrichment analysis indicated that ubiquitin-mediated proteolysis and proteasome pathways may play essential roles in the formation and development of HNSCC (Figure 2D).

### Construction and identification of the prognostic risk model

To identify the prognostic value of DEUPSGs in HNSCC patients, Univariate Cox regression analysis was used to confirm the expression patterns of 114 DEUPSGs in the TCGA training set. The forest plots displayed the eight prognosis-related DEUPSGs in HNSCC (Figure 1C). The prognostic risk model was established based on the eight prognosis-related DEUPSGs using LASSO regression analysis (Supplementary Figure 2). The coefficient values of the eight DEUPSGs were shown in Table 1. The risk score was calculated for each sample as the following equation:

Risk score = BRCA1 * (-0.037) + OSTM1 * 0.0294 + PCGF2 * 0.0502 + PSMD2 * 0.0058 + SOCS1 * (-0.0157) + UCHL1 * 0.0022 + UHRF1 * (-0.0496) + USP54 * (-0.0887)

**Table 1:** List of the eight prognostic genes of the risk model in the TCGA training set.

**ENSG ID**	**Symbol**	**Location**	**Expression status**	**Coefficient**
ENSG00000012048	BRCA1	Chromosome 17	Upregulated	-0.0370
ENSG00000081087	OSTM1	Chromosome 10	Upregulated	0.0294
ENSG00000277258	PCGF2	Chromosome 17	Upregulated	0.0502
ENSG00000175166	PSMD2	Chromosome 3	Upregulated	0.0058
ENSG00000185338	SOCS1	Chromosome 16	Upregulated	-0.0157
ENSG00000154277	UCHL1	Chromosome 4	Upregulated	0.0022
ENSG00000276043	UHRF1	Chromosome 19	Upregulated	-0.0496
ENSG00000166348	USP54	Chromosome 10	Downregulated	-0.0887

**Figure 3:**
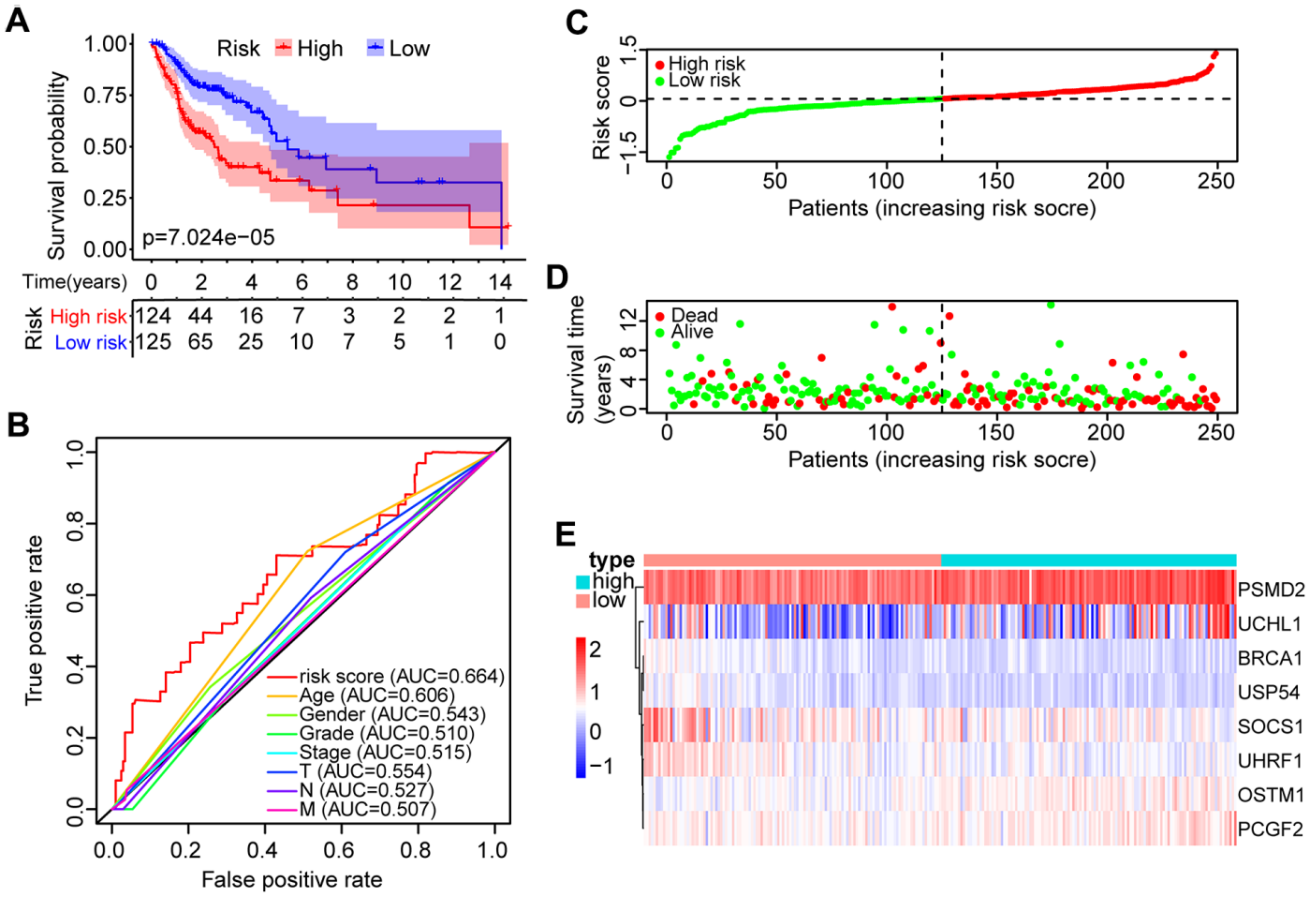
**Identification of the prognostic risk model in HNSCC patients.** (**A**) Kaplan-Meier survival curve with overall survival (OS) in the high- and low-risk HNSCC patients in the TCGA training set. (**B**) ROC curve showing AUC for the risk score and other clinical factors of HNSCC patients in the TCGA training set. (**C**) The risk plot distribution of the high- and low-risk HNSCC patients. (**D**) Scatter plot showing the survival status of HNSCC patients. (**E**) The expression of risk genes of HNSCC samples in the TCGA training set.

Samples were classified into two groups based on the median risk score (0.0622) of the training set. OS analysis indicated that the low-risk group has a markedly better prognosis than the high-risk group (*P* < 0.0001, Figure 3A). The receiver operating characteristic (ROC) curve indicated that the area under the ROC (AUC) value was 0.664 (Figure 3B). The risk scores and OS were performed by risk plots and scatter plots (Figure 3C, 3D). Expression patterns of risk genes in the high- and low-risk groups were depicted in Figure 3E, showing that high expression levels of PSMD2, OSTM1 (Osteoclastogenesis associated transmembrane protein 1), PCGF2 (polycomb group ring finger 2), and UCHL1 (ubiquitin C-terminal hydrolase L1) can be considered as risk factors were correlated with a high-risk score. Furthermore, high expression levels of BRCA1 (BRCA1 DNA repair associated), SOCS1 (suppressor of cytokine signaling 1), UHRF1 (ubiquitin-like with PHD and ring finger domains 1), and USP54 (ubiquitin specific peptidase 54) were associated with a low-risk score.

An independent validation data set was involved in the evaluation of the risk model. Based on the prognostic risk model, patients with HNSCC were divided into low- and high-risk groups. Survival analysis performed by Kaplan-Meier displayed significant prognostic differences between the two groups (*P* < 0.001, Supplementary Figure 3A). The relationship between the eight DEUPSGs and risk scores was shown in Supplementary Figure 3B. At the same time, similar results were found in the TCGA training set and TCGA all data set (Supplementary Figure 3C, 3D).

The GEO (GSE65858) database was used as an external data set for validating the classification performance of the risk model. Samples in the GSE65858 were segregated into low- and high-risk groups. The classification performance of the risk model and the expression pattern of the risk genes were consistent with the training set (Supplementary Figure 4).

### Clinical independence of the risk model

**Figure 4:**
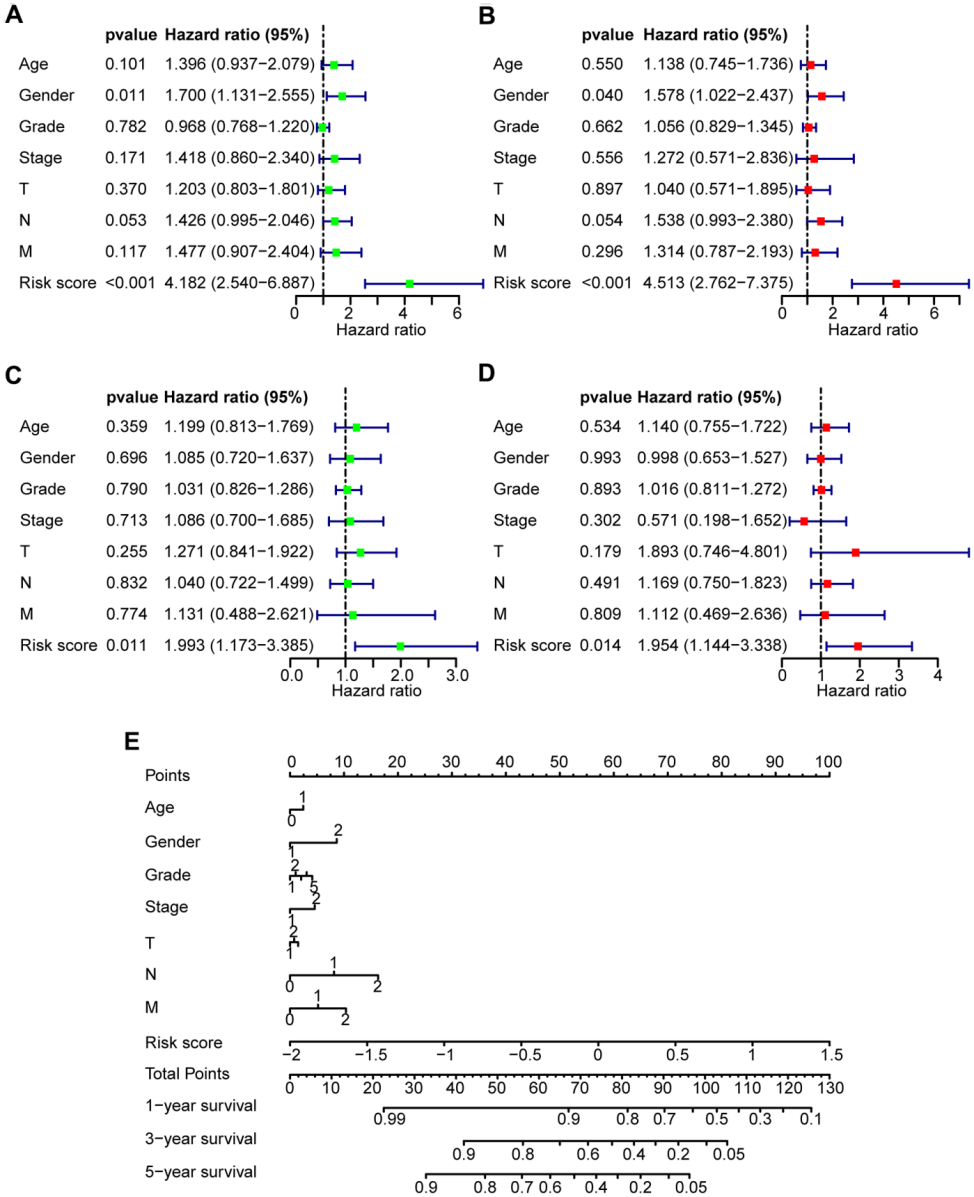
**Prognostic predictive value of risk score in HNSCC patients.** (**A**, **B**) Univariate and multivariate Cox regression analyses of the clinical factors of the patients in the TCGA training set, respectively. (**C**, **D**) Univariate and multivariate Cox regression analyses of the clinical factors of the patients in TCGA test set, respectively. (**E**) Nomogram for OS in HNSCC patients.

To assess the independence of the risk model in clinical application, univariate and multivariate Cox regression analyses were subjected to clinical parameters from the TCGA training set, TCGA test set, GSE65858 database, and TCGA all data set. Univariate and multivariate Cox regression analyses suggested that the risk score had a significant association with prognosis in the TCGA training set (HR = 4.182, 95% CI = 2.540-6.887, *P* < 0.001; HR = 4.513, 95% CI = 2.732-7.375, *P* < 0.001, respectively, Figure 4A, 4B), the TCGA test set (HR = 1.993, 95% CI = 1.173-3.385, *P* < 0.05; HR = 1.954, 95% CI = 1.144-3.338, *P* < 0.05, respectively, Figure 4C, 4D), the TCGA all data set (HR = 2.947, 95% CI = 2.048-4.241, *P* < 0.01; HR = 2.961, 95% CI = 2.051-4.277, *P* < 0.01, respectively, (Supplementary Figure 5A, 5B), and the GEO data set (HR = 1.754, 95% CI = 0.917-3.352, *P* < 0.01; HR = 1.598, 95% CI = 0.814-3.135, *P* < 0.05, respectively, Supplementary Figure 5C, 5D). These data demonstrated that the risk model has an effectively prognostic power and exhibits an independent predictive value. A nomogram contained both risk score and clinical features was shown in Figure 4E.

### Gene set enrichment analysis in the high-risk and low-risk groups

**Figure 5:**
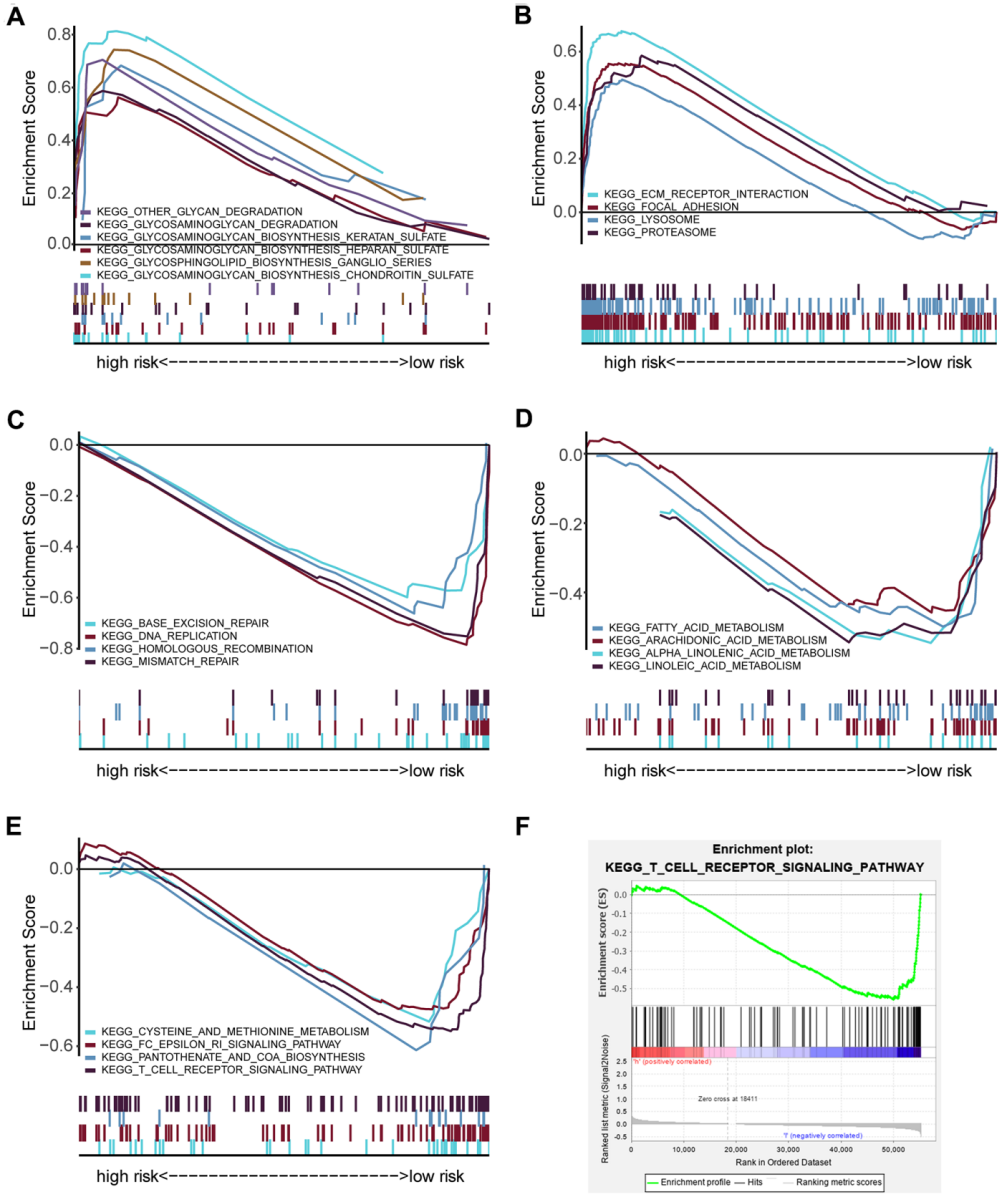
**GSEA analysis showing the enriched pathways of the high- and low-risk groups.** (**A**) Multiple GSEA showing glycan-related metabolism pathways in the high-risk group. (**B**) Multiple GSEA showing extracellular matrix and proteolysis related pathways in the high-risk group. (**C**) Multiple GSEA showing DNA repair in the low-risk group. (**D**) Multiple GSEA showing fatty acid metabolism pathways in the low-risk group. (**E**) Multiple GSEA showing other metabolism- and immune- related pathways in the low-risk group. (**F**) Single GSEA showing the T cell receptor signaling pathway.

Gene set enrichment analysis (GSEA) was employed for pathways identification in the TCGA database for detecting twenty pathways from the high- and low-risk groups (Supplementary Table 3). The selected signaling pathways showed significant differences between the two groups (FDR < 0.25, NOM *p* < 0.05) (Table 2), in which glycan metabolism, extracellular matrix, and proteolysis were significantly enriched in the high-risk group (Figure 5A, 5B). In contrast, DNA repair, fatty acid metabolism, other metabolisms, and immune-related pathways were significantly enriched in the low-risk group (Figure 5C–5E). Interestingly, the T cell receptor (TCR) signaling pathway was enriched in the low-risk group (Figure 5F), which may indicate a potential relationship of the high-risk core with impaired immunosurveillance and T cell antitumor response in HNSCC. The rest of GSEA graphs were shown in Supplementary Figure 6.

**Table 2:** Gene sets enriched in high-risk and low-risk groups.

**MSigDB collection**	**Name**	**NES**	**ES**	**NOM *p*-val**	**FDR *q*-val**
c2.cp.kegg.v7.1.symbols.gmt	KEGG_GLYCOSAMINOGLYCAN_BIOSYNTHESIS_CHONDROITIN_SULFATE	2.099	0.813	0.000	0.010
	KEGG_ECM_RECEPTOR_INTERACTION	1.903	0.676	0.010	0.061
	KEGG_GLYCOSPHINGOLIPID_BIOSYNTHESIS_GANGLIO_SERIES	1.889	0.743	0.002	0.046
	KEGG_OTHER_GLYCAN_DEGRADATION	1.854	0.705	0.006	0.038
	KEGG_FOCAL_ADHESION	1.813	0.556	0.018	0.045
	KEGG_GLYCOSAMINOGLYCAN_BIOSYNTHESIS_KERATAN_SULFATE	1.801	0.683	0.004	0.045
	KEGG_GLYCOSAMINOGLYCAN_DEGRADATION	1.758	0.587	0.008	0.052
	KEGG_LYSOSOME	1.746	0.495	0.018	0.047
	KEGG_GLYCOSAMINOGLYCAN_BIOSYNTHESIS_HEPARAN_SULFATE	1.719	0.563	0.010	0.054
	KEGG_PROTEASOME	1.634	0.585	0.047	0.120
	KEGG_DNA_REPLICATION	-1.971	-0.810	0.000	0.143
	KEGG_MISMATCH_REPAIR	-1.961	-0.796	0.004	0.081
	KEGG_T_CELL_RECEPTOR_SIGNALING_PATHWAY	-1.917	-0.559	0.004	0.065
	KEGG_PANTOTHENATE_AND_COA_BIOSYNTHESIS	-1.841	-0.669	0.002	0.126
	KEGG_HOMOLOGOUS_RECOMBINATION	-1.803	-0.690	0.016	0.148
	KEGG_CYSTEINE_AND_METHIONINE_METABOLISM	-1.792	-0.552	0.012	0.137
	KEGG_FC_EPSILON_RI_SIGNALING_PATHWAY	-1.773	-0.488	0.008	0.141
	KEGG_ALPHA_LINOLENIC_ACID_METABOLISM	-1.726	-0.610	0.010	0.153
	KEGG_LINOLEIC_ACID_METABOLISM	-1.712	-0.565	0.018	0.155
	KEGG_BASE_EXCISION_REPAIR	-1.676	-0.620	0.045	0.152
	KEGG_ARACHIDONIC_ACID_METABOLISM	-1.667	-0.476	0.014	0.152
	KEGG_FATTY_ACID_METABOLISM	-1.660	-0.527	0.028	0.151

### Exploration of the relationship between the risk score and antitumor immunity

**Figure 6:**
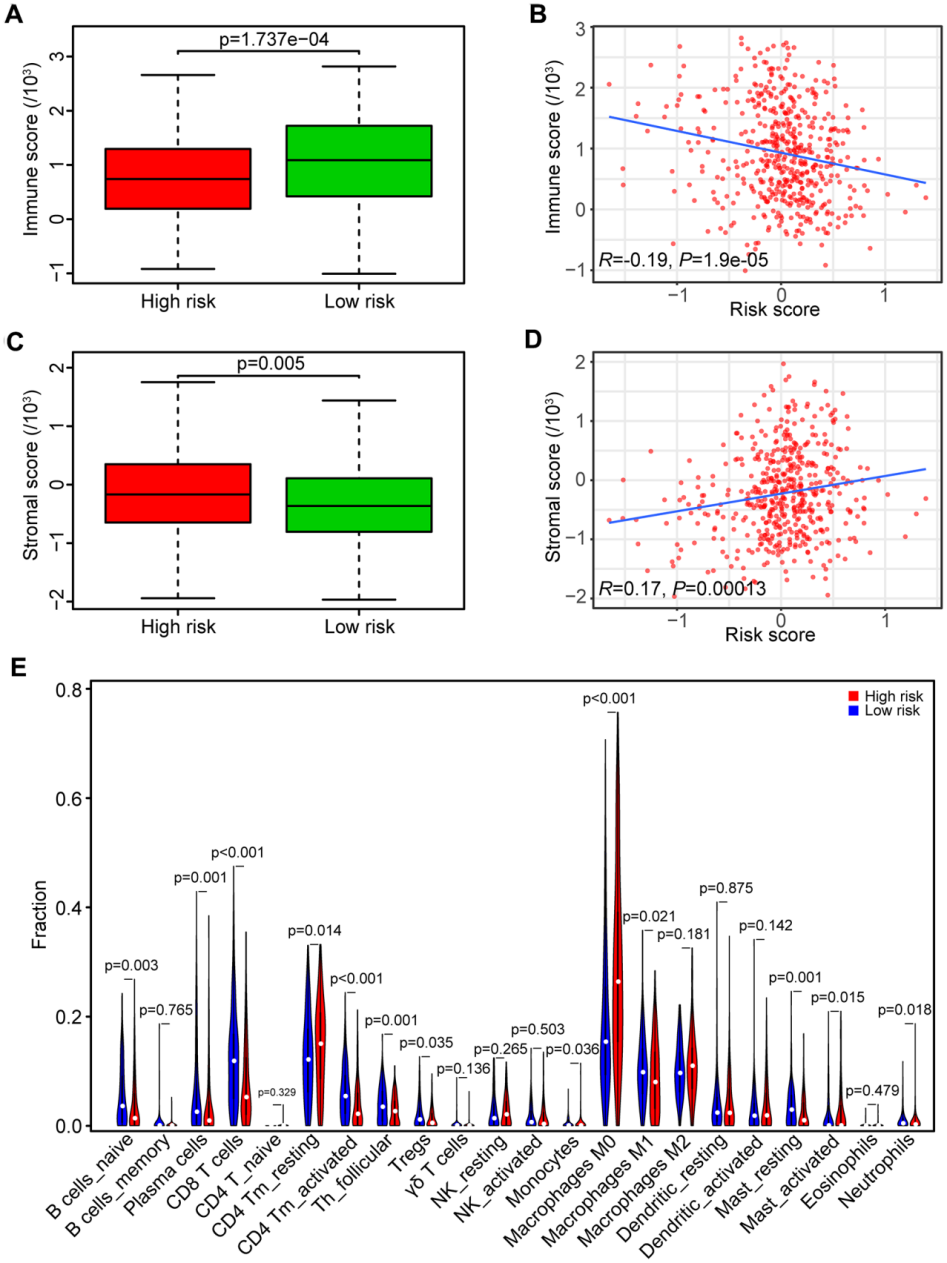
**Association between risk score and tumor immunity.** (**A**) Distribution of immune scores in high- and low-risk HNSCC patients. (**B**) Association between the risk score and immune score in HNSCC samples. (**C**) Distribution of stromal scores in high- and low-risk HNSCC patients. (**D**) Association between the risk score and stromal score in HNSCC samples. (**E**) Comparison of immune cell fractions between the high-risk and low-risk HNSCC patients.

To determine whether the high-risk score was correlated with impaired T cell activity in HNSCC, we employed the ESTIMATE algorithm to estimate immune and stromal scores based on the TCGA database. The immune score was higher in the low-risk group than the high-risk group (*P* < 0.001, Figure 6A), and the risk score had a negative correlation with the immune score in HNSCC patients (*R* = -0.19, *P* < 0.0001, Figure 6B). However, the stromal score was higher in the high-risk group than the low-risk group (*P* < 0.01, Figure 6C), and the risk score had a positive correlation with the stromal score in HNSCC samples (*R* = 0.17, *P* < 0.001, Figure 6D).

We subsequently analyzed the fraction of both tumor-infiltrated innate and adaptive immune cells in HNSCC samples by CIBERSORT. The relationships of risk score with those immune cells were shown in Figure 6E. The results showed that the fraction of CD8 T cells (*P* < 0.001), CD4 memory activated T cells (*P* < 0.001), and follicular helper T cells (*P* < 0.01) in samples from the high-risk group were lower than the low-risk group, indicating that high-risk score was associated with immunosuppressive phenotypes that may result from impaired T cell response and activation.

### Correlation between the genes of the risk model and the four T cell subpopulations

**Figure 7:**
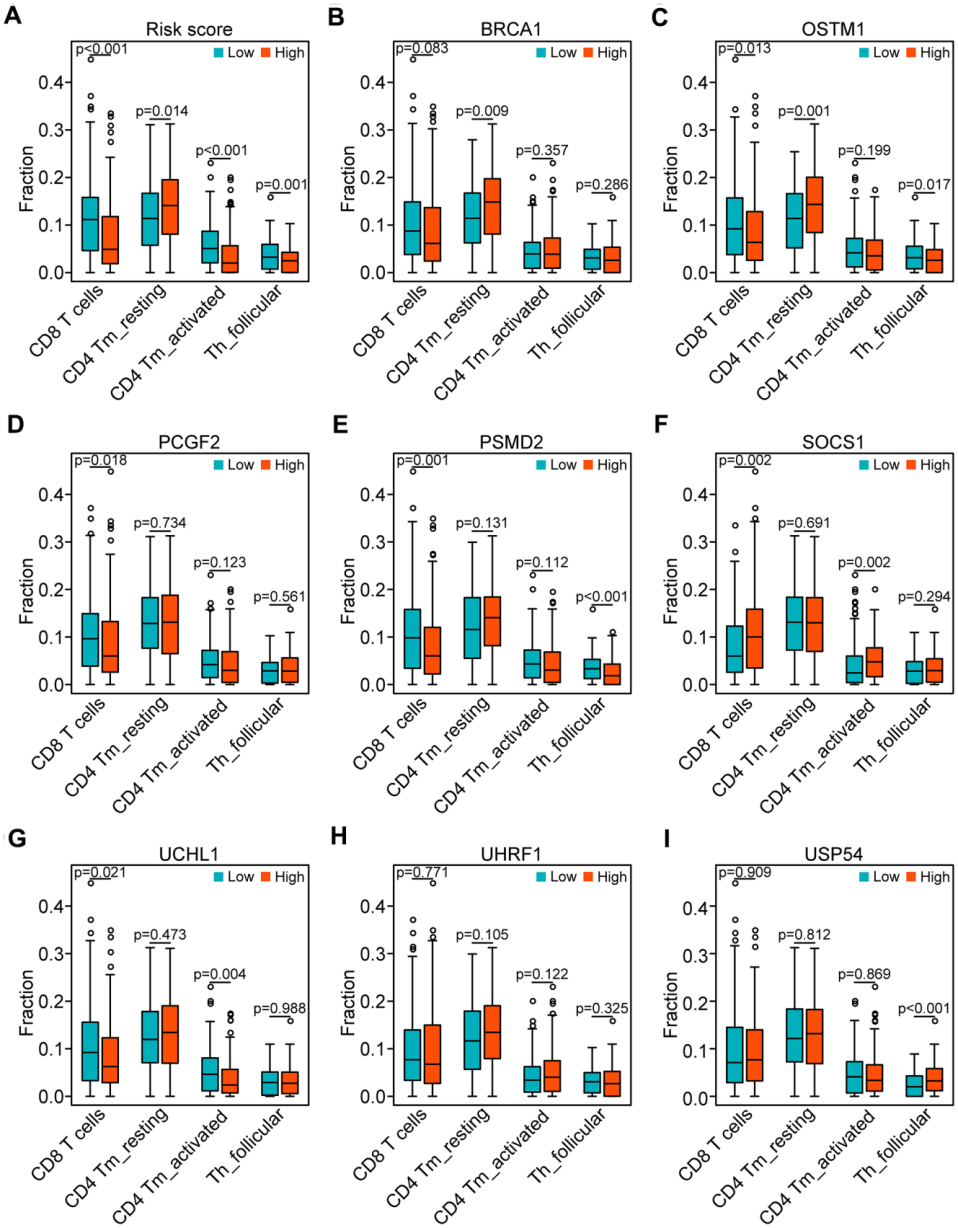
**Correlation of the genes of the risk model with the four subpopulations of T cells.** (**A**) Comparison of the four subpopulations of T cells (CD8 T cells, CD4 memory activated T cells, and follicular helper T cells) between the high- and low-risk groups. (**B**–**I**) Distribution of the four T cell subpopulations based on the high and low expression of BRCA1, OSTM1, PCGF2, PSMD2, SOCS1, UCHL1, UHRF1, and USP54, respectively.

According to the potential relationship between the risk model and the four T cell subpopulations (Figure 7A), we further tested the correlation between the eight genes and the four T cell subpopulations (Figure 7B–7I). Consistent with the profile of these genes in the risk model, decreased CD8 T cells were associated with the upregulated OSTM1 (*P* < 0.01), PCGF2 (*P* < 0.05), PSMD2 (*P* < 0.01), UCHL1 (*P* < 0.05) and the downregulated SOCS1 (*P* < 0.01). Additionally, decreased CD4 memory-activated T cells were related to the upregulated UCHL1 (*P* < 0.01) and the downregulated SOCS1 (*P* < 0.01). Similarly, the asthenia of follicular helper T cells may be caused by high expressed OSTM1 (*P* < 0.05), PSMD2 (*P* < 0.001) and low expressed USP54 (*P* < 0.001). Moreover, the increase of CD4 memory resting T cells was associated with the high expressed OSTM1 (*P* < 0.01). Thus, the UPS-related genes OSTM1, PCGF2, PSMD2, UCHL1, SOCS1, and USP54 were associated with immunosuppression in HNSCC.

## DISCUSSION

Tumor cells that escape from immunosurveillance and attack have been the characteristics of immunosuppression in the tumor progression, which is largely associated with the impaired or dysregulated host immune system [27]. By managing protein degradation and abundance, the UPS pathway has been reported as an essential regulator in immunosurveillance of the tumor microenvironment [28]. Accumulating evidence has demonstrated that E3 dysfunction perturbs antitumor immune responses [29–31]. Current knowledge on the functions of the UPS pathway in the development of HNSCC remains obscure. In this regard, an improved understanding of the UPSGs functions in the text of HNSCC can provide a therapeutic value.

This study analytically conferred prognostic evidence by identification of 114 DEUPSGs and establishment of the eight prognosis-related DEUPSGs. A prognosis risk model was established based on the eight DEUPSGs. Previous studies have reported the abnormal expression of UPSGs in renal cancer and breast cancer [32, 33]. Analyses performed at both mRNA and protein levels can improve the reliability of the results, whereas the limited sample size of these analyses may lead to a bias in the results, because only abnormal gene expression is analyzed, and verification of clinical significance or potential functional analysis is lacking.

In this risk model, high expression levels of PSMD2, OSTM1, PCGF2, and UCHL1 were risk factors, while high expression levels of BRCA1, SOCS1, UHRF1, and USP54 acted as protective factors. Overexpression of PSMD2 has been previously shown to be involved in the development of lung adenocarcinoma, breast cancer, and hepatocellular carcinoma [24–26]. UCHL1 has been reported a potential oncoprotein in colorectal cancer, breast cancer, and uterine serous cancer. It promotes proliferation and metastasis of cancer cells and leads to a radioresistant phenotype via regulation of β-catenin/TCF and HIF-1α pathway [34–38]. In line with those previous studies, our results also suggested that PSMD2 and UCHL1 may have therapeutic potential as targets against cancer. Accumulating evidence has shown that BRCA1 mutation is a crucial risk factor in multiple cancers, including breast cancer, skin cancer, ovarian cancer, and colorectal cancer [39–43]. SOCS1 acts as an antioncogene in various tumors, arresting the cell cycle, inhibiting cancer cell migration and invasion, and attenuating tumor growth [44–47]. Nevertheless, non-coding RNA (ncRNA) regulating SOCS1 promotes the occurrence and development of cancers [48–50]. Similarly, UHRF1 also acts as an antioncogene in multiple cancers and inhibits tumor development and progression [51–54]. Therefore, the abovementioned studies suggest that BRCA1, SOCS1, and UHRF1 serve as antioncogenes, consistent with our results.

Interestingly, our GSEA results showed that the TCR signaling pathway was highly enriched in the low-risk group, which suggested the impaired T cell activation presented in the high-risk group. TCR engagement initiates a central signaling pathway that is crucial for T cell proliferation, survival, and differentiation into killer cells for adaptive immunity. Abnormalities of TCR signaling could result in immunodeficiency [55–58]. Consequently, according to the relationship between the risk score and the distribution of T cell subpopulations, we found that the high-risk score has a decreased immune score, which may suggest that the high-risk score is critical for evaluating whether tumor-infiltrated T cells have an immunosuppressive status and dysregulated antitumor response. Moreover, the high expression of OSTM1, PCGF2, PSMD2 combined with the low expression of UCHL1, SOCS1, and USP54 were engaged in the suppression of T cell proliferation and activation in the HNSCC microenvironment. It has been previously shown that differential expression of UCHL1 was majorly involved in the proliferation and differentiation of T cells, including CD8 memory CTLp, circulating TCR-gamma/delta+ lymphocytes, and mature T cells [59]. SOCS1 has been identified as an inhibitor against cytokine release and a growth factor receptor, it plays a crucial role in regulating T cell homeostasis, development, and homeostasis activation [60–64]. However, the function of OSTM1, PCGF2, PSMD2, and USP54 have not been investigated in the antitumoral immune response. The present studies suggest that the high expression of OSTM1, PCGF2, and PSMD2 and low expression of UCHL1 were associated with immunosuppressive phenotypes.

Generally, we identified an eight -UPSGs based risk model according to the TCGA-HNSCC database and analyzed its biological functions, demonstrating that the risk score obtained from this model was significantly correlated with immunosuppressive status. However, some limitations in the context of the study should be acknowledged. First, this study is performed by bioinformatics analysis alone, the predictive results could be insufficient. Second, this is a retrospective study rather than a prospective one, further validations with large clinical cohort and actual experiments are needed.

## CONCLUSIONS

In summary, we present a risk model constructed by DEUPSGs that can be considered as potential prognostic biomarkers and associated with an immunosuppressive status and impaired antitumor response of T cells in the HNSCC microenvironment. This systematic analysis on the interaction of UPSGs based risk model and immune profile provides a novel understanding of the precise immunotherapy for HNSCC patients.

## MATERIALS AND METHODS

### Data downloading and processing

The most recent transcriptional data and clinical features on 500 HNSCC samples and 44 adjacent samples were obtained from the TCGA database (https://portal.gdc.cancer.gov/) and the cBio Cancer Genomics portal (https://www.cbioportal.org/) (Table 3). We chose 498 HNSCC samples with follow-up data and randomly divided them into two groups: a training set (n=298) and a test set (n=298), as shown in Supplementary Tables 4, 5. In addition, we obtained the GSE65858 data setwith 270 HNSCC samples as an external set from the Gene Expression Omnibus (GEO) database (https://www.ncbi.nlm.nih.gov/geo/).

**Table 3:** Clinical characteristics of HNSCC patients in the TCGA and GEO databases.

**Clinical characteristics**	**TCGA**			**GEO (GSE65858)**	
	**n=500**	**%**		**n=270**	**%**
**Age**					
< 60	220	44.0		153	56.7
≥ 60	280	56.0		117	43.3
**Gender**					
Female	133	26.6		47	17.4
Male	367	73.4		223	82.6
**Histologic grade**					
G1	61	12.2			
G2	299	59.8			
G3	119	23.8			
G4	2	0.4			
Gx	16	3.2			
NA	3	0.6			
**Stage**					
I	19	3.8		18	6.7
II	95	19.0		37	13.7
III	102	20.4		37	13.7
IV	270	54.0		178	65.9
NA	14	2.8			
**T classification**					
T1	33	6.6		35	13
T2	143	28.6		80	29.6
T3	130	26.0		58	21.5
T4	179	35.8		97	35.9
Tx	11	2.2			
NA	4	0.8			
**N classification**					
N0	239	47.8		94	34.8
N+	239	47.8		176	65.2
Nx	18	3.6			
NA	4	0.8			
**M classification**					
M0	470	94.0		263	97.4
M1	5	1.0		7	2.6
Mx	20	4.0			
NA	5	1.0			
**Vital status**					
Deceased	218	43.6		94	34.8
Living	282	56.4		176	65.2

### Identification of differentially expressed genes

The differentially expressed UPS-related genes (DEUPSGs) between head and neck tumor tissues and adjacent tissues were screened out through the Wilcoxon signed-rank test using the limma R package, according to the following cut-off values: FDR < 0.05 and |Fold change| > 1.5. Subsequently, a heat map was generated using the “pheatmap” package, and volcano dot plots were created to present the DEUPSGs. We used the OmicCircos R package to show the distribution of the DEUPSGs [65].

### Functional enrichment analyses

The enrichplot R package was built for the GO and KEGG pathway enrichment analyses to identify the functions of the abovementioned DEUPSGs [66], containing BP, CC, and MF terms and pathways. Statistical significance was defined as both *P*-value and FDR < 0.05.

### Prognostic risk model establishment

We used univariate Cox regression analysis to screen out DEUPSGs significantly associated with OS. *P* < 0.05 was chosen as the threshold. We further used Lasso regression analysis to establish a multi-gene prognostic model. The risk score of each patient was calculated, employing the regression coefficient of each selected gene based on the following equation:

$$Risk\;score=\sum_{i=1}^{n}Coef\;(gene_{i})*Exp_{i},$$

where n is the number of prognostic genes, *gene_i_* is the *_i_*th prognostic gene, *Coef* is the regression coefficient of genes, and *Exp_i_* is the expression value of the prognostic genes. Then, the HNSCC patients were divided into high-risk and low-risk groups according to the median risk score. Moreover, we used *rms* package to generate a nomogram.

### Gene set enrichment analysis

Gene set enrichment analysis (GSEA, https://www.gsea-msigdb.org/) is a powerful computational tool used to determine statistical differences of specific functional gene sets between two biological states [67]. Here, we performed the GSEA analysis using GSEA v4.0.3 to obtain the differentially expressed genes from the high- and low-risk groups. After 1000 repeats for each analysis, gene sets with *p-*value < 0.05 and FDR < 0.25 were identified as enriched sets.

### Identification of immune scores and tumor-infiltrating immune cells

To identify infiltrated immune cell numbers and stromal cell numbers, the R software package “ESTIMATE algorithm” was used for calculating immune and stromal scores of HNSCC samples in the TCGA database [68]. Additionally, CIBERSORT was applied to acquire the composition of the infiltrated immune cells in HNSCC samples [69].

### Statistical analysis

R package (v4.0.1) was subjected to all analyses. Kaplan-Meier curve was drawn with the log-rank test. Univariate and multivariate Cox regression was used to determine the independence of gene markers. Spearman’s rank correlation test was utilized to identify the variables. Differences in the distributions of the variables were analyzed by the Chi-square test or Fisher’s exact test. *P* < 0.05 was considered statistically significant.

### Data availability statement

Expression profiles of data sets in this study were downloaded from The Cancer Genome Atlas (TCGA) (https://portal.gdc.cancer.gov/) and Gene Expression Omnibus (GEO) database (https://www.ncbi.nlm.nih.gov/geo/).

## Supplementary Material

Supplementary Figures

Supplementary Table 1

Supplementary Table 2

Supplementary Table 3

Supplementary Table 4

Supplementary Table 5
