# Supplementary material for: Comprehensive analysis of ubiquitin-proteasome system genes related to prognosis and immunosuppression in head and neck squamous cell carcinoma
Source: Aging (Albany NY). 2021 Aug 16;13(16):20277–301. doi: 10.18632/aging.203411 (PMC8436932; doi:10.18632/aging.203411)
Supplement: Supplementary Figures [file aging-13-203411-s001.pdf]

SUPPLEMENTARY FIGURES

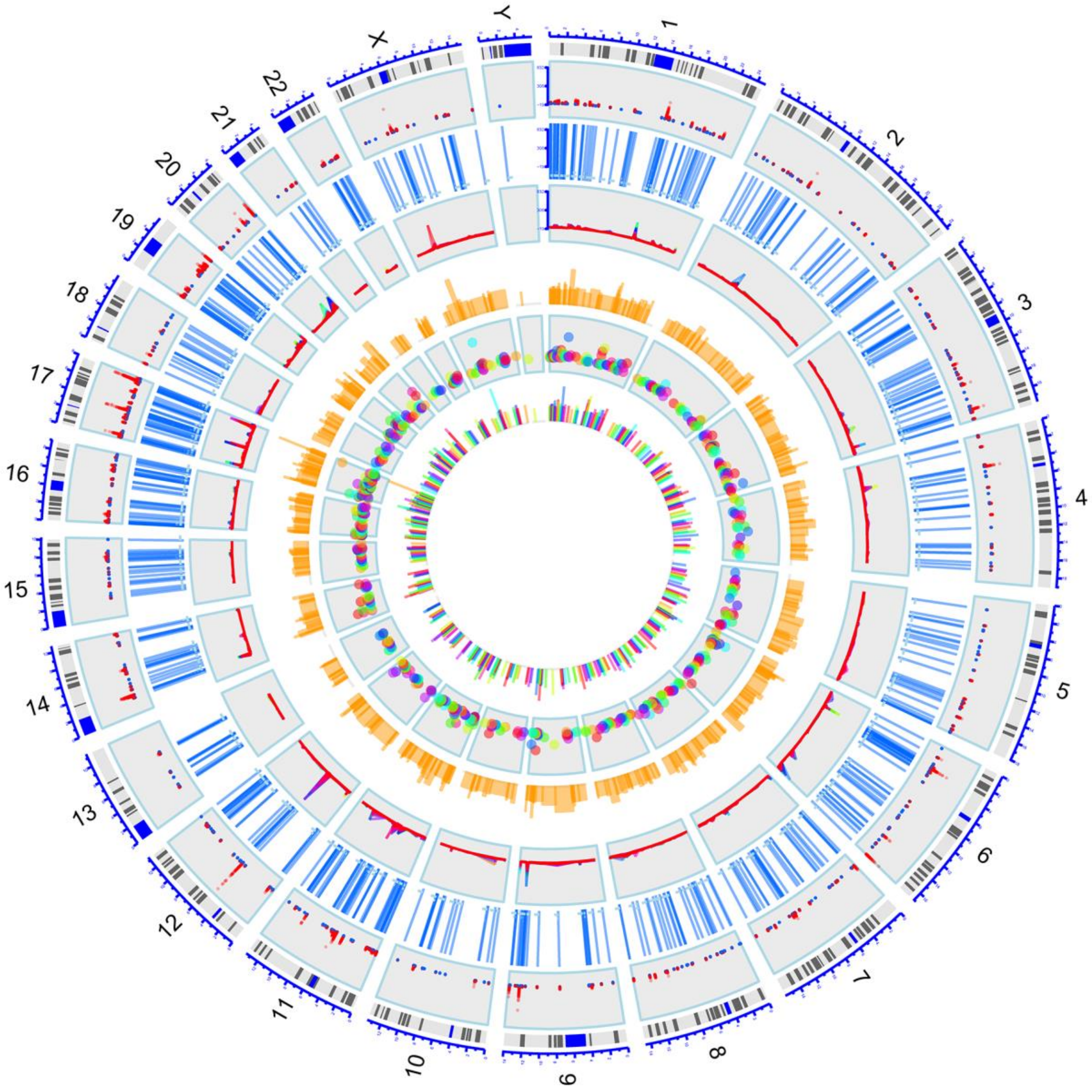

Supplementary Figure 1. The distribution of UPSGs on chromosomes. A total of 804 human UPSGs are distributed across all chromosomes, including the sex chromosome X and Y. The position of the line and dot indicates the position of the gene on the chromosome, and the height of the column indicates the abundance of gene expression.

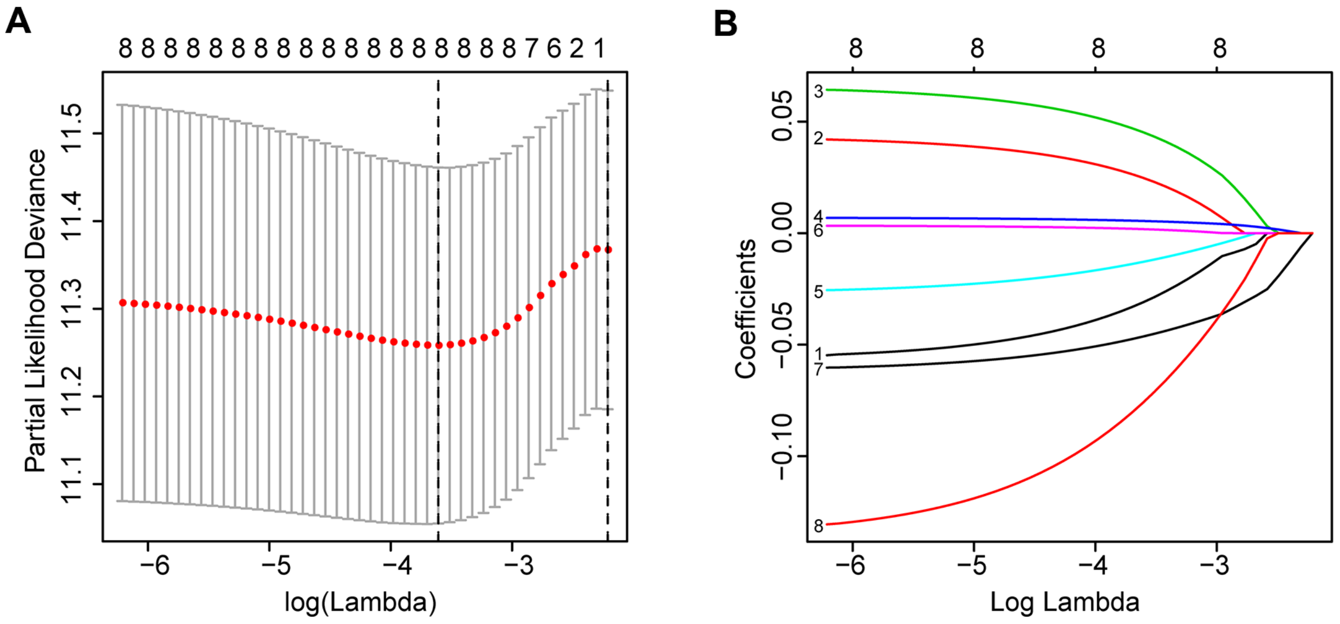

Supplementary Figure 2. Construction of a prognostic risk model using LASSO regression analysis. (**A**) LASSO algorithms used to identify prognosis-related DEUPSGs. (**B**) The prognostic risk model constructed using LASSO coefficient values in the TCGA training set.

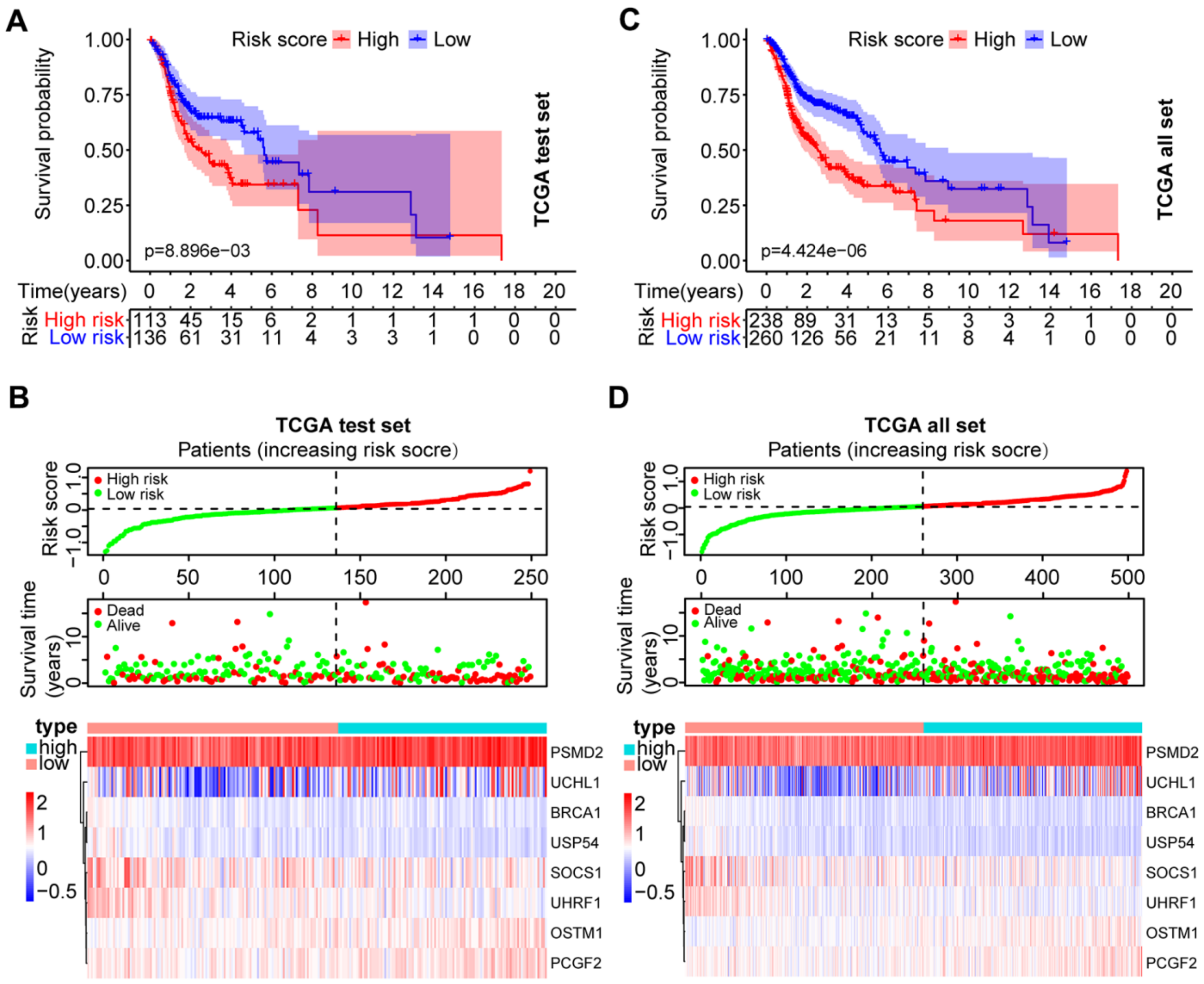

Supplementary Figure 3. Verification of the prognostic risk model in HNSCC patients in the TCGA database. (**A**) Kaplan-Meier survival curve for HNSCC patients in the TCGA test set. (**B**) Kaplan-Meier survival curve for HNSCC patients in the TCGA all set. (**C**) The risk plot distribution, survival status, and the expression of risk genes of HNSCC patients in the TCGA test set. (**D**) The risk plot distribution, survival status, and expression of risk genes in HNSCC patients in the TCGA all set.

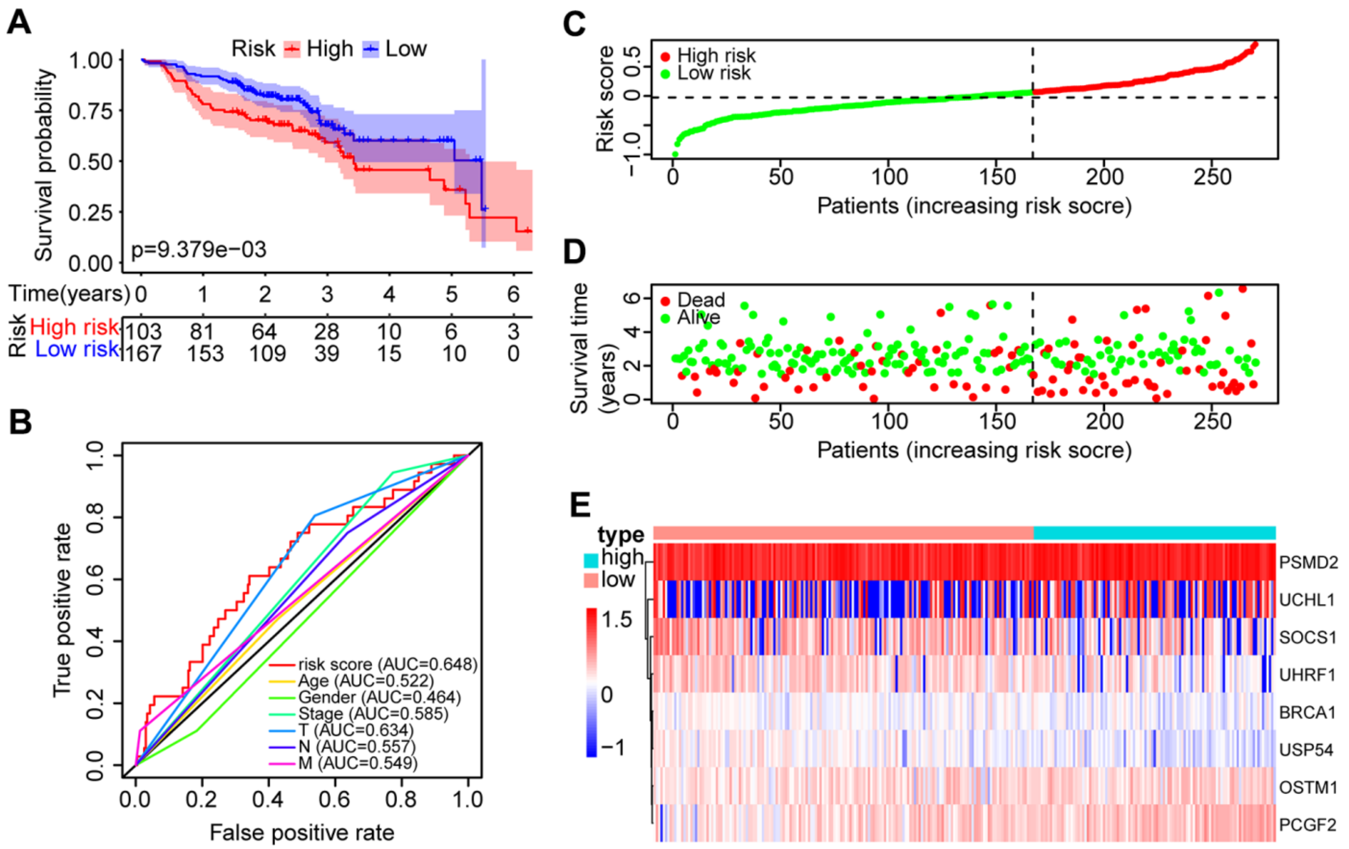

Supplementary Figure 4. Performance validation of the risk model in HNSCC patients using the GEO database. (**A**) Kaplan-Meier survival curve with OS in the high- and low-risk HNSCC patients in the GSE65858 data set. (**B**) ROC curve showing the AUC for the risk score and other clinical factors of HNSCC patients in the GEO database. (**C**) The risk score distribution of the high- and low-risk HNSCC patients. (**D**) Scatter plot showing the survival statuses of HNSCC patients. (**E**) The expression of risk genes of HNSCC samples in the GSE65858 data set.

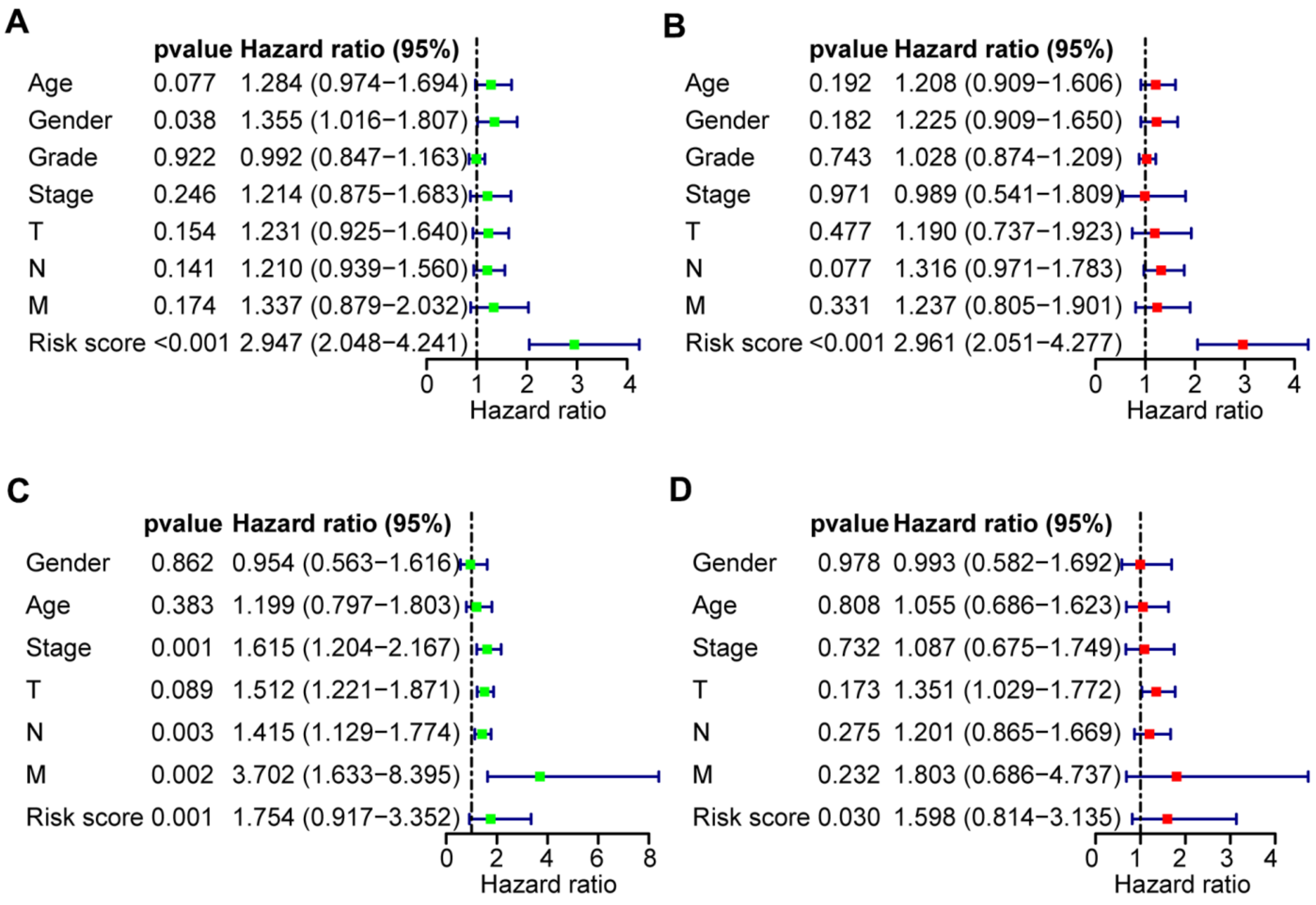

Supplementary Figure 5. Univariate and multivariate Cox regression analyses of OS in the GEO and TCGA data sets. (**A**, **B**) The clinical factors of the patients in the TCGA all set were assessed using univariate and multivariate Cox regression analysis, respectively. (**C**, **D**) The clinical factors of the patients in GEO (GSE65858) data set were assessed using univariate and multivariate Cox regression analysis, respectively.

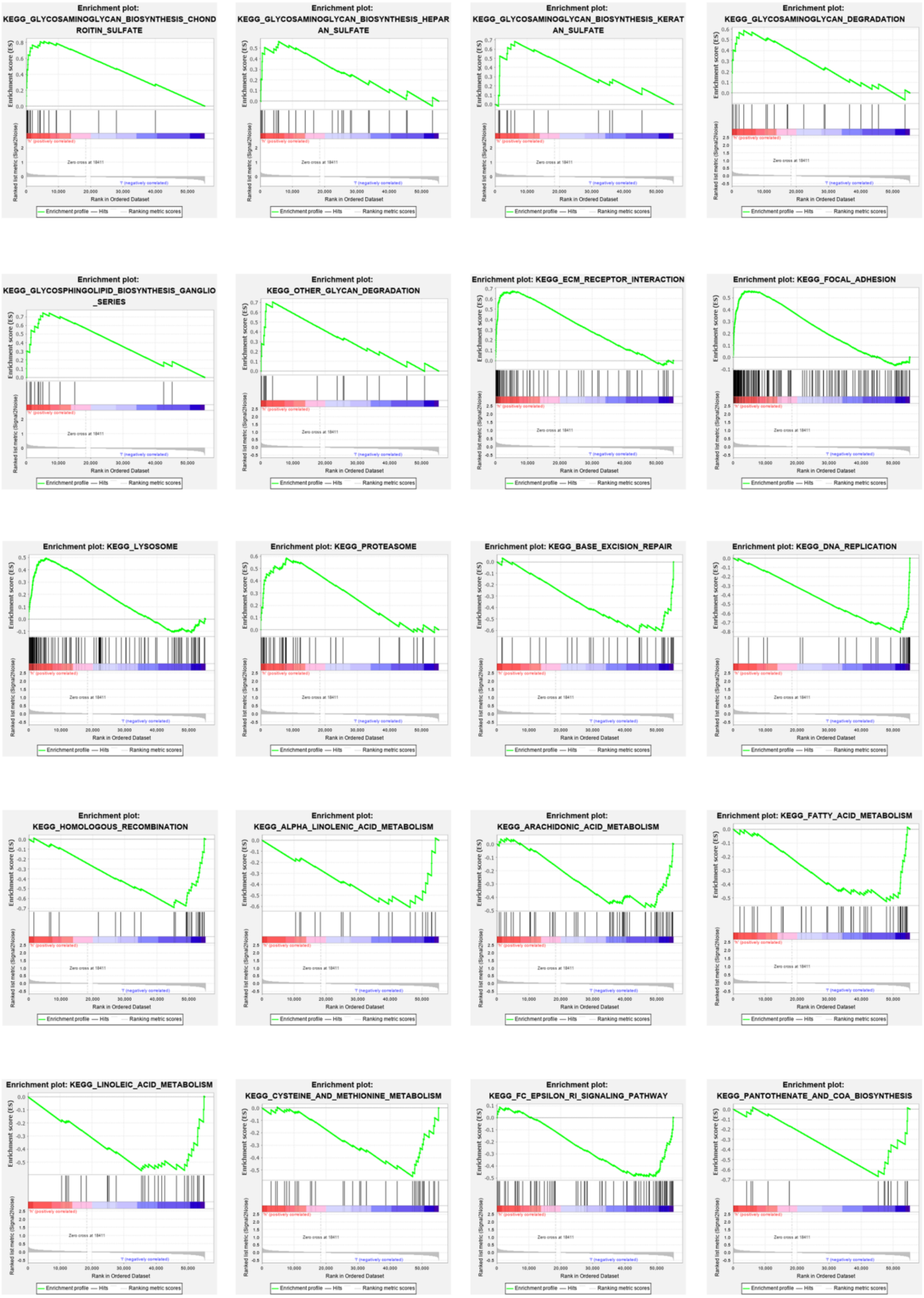

Supplementary Figure 6. Other single GSEA figures of the high-risk and low-risk groups. Single GSEA plots showing enriched pathways in the high- and low-risk groups displayed in Table 3.
